# Editorial: Cancer and bone metastasis, volume II

**DOI:** 10.3389/fendo.2022.971240

**Published:** 2022-07-21

**Authors:** Chandi C. Mandal, Julie A. Rhoades (Sterling)

**Affiliations:** ^1^ Department of Biochemistry, Central University of Rajasthan, Ajmer, India; ^2^ Tennessee Valley Healthcare System, Department of Veterans Affairs, Nashville, TN, United States; ^3^ Department of Medicine, Vanderbilt University Medical Center, Nashville, TN, United States

**Keywords:** cancer, bone metastases (BM), risk factors, cell signaling, therapy

Enormous efforts in basic and clinical research in bone metastatic disease have led to major enhancements in patient quality of lives. However, these advances have yet to significantly prolong survival and have not cured disease and patients with bone metastatic disease will eventually succumb to disease. Bone metastatic diseases have become more clinically significant as the treatments of the primary tumor have improved, which lead to a renewed focus on treating the tumors that reside in bone. The tumor types with the highest incidence of bone metastatic disease are some of the most common tumors world-wide, including breast, prostate, and lung. Other tumors that frequently invade or metastasize to bone include, multiple myeloma, melanoma, renal cancer, and head and neck tumors. These metastases often cause bone degradation *via* excessive activation of osteoclast-driven osteolysis. The structural deformities lead to develop nerve compression, pain, fractures and morbidity to the bone metastatic cancer patients. However, treatment modalities available for such bone diseases are not curative. Thus, understanding the in-depth molecular mechanisms and finding risk factors are the urgent need for the treatment of deadliest bone metastasis (see previous topic: Cancer and Bone Metastasis (1). In this context, articles published on the topic “Cancer and Bone Metastasis Volume II” have described the prediction of risk factors and cellular interaction involved in bone metastasis and bone diseases along with some possible therapies for these diseases.

## Risk factor analysis for bone metastasis

One of the remaining challenges to treating bone metastatic disease is the lack of models that can help predict which patients may develop bone metastatic disease and how patients may respond to therapy. Thus, developing novel approaches for risk factor assessment remain a critical need.

Chen et al. research group suggested based on a population-based study that various risk factors and prognostic factors along with nomograms could be used for personalized prognosis prediction of distant metastasis (DM) for osteosarcoma patients through analysis of data of osteosarcoma patients diagnosed between 2004-2015 based on the results obtained in ROC, calibration and Kaplan-Meier- survival analysis. Moreover, osteosarcoma patients with DM predicted by the nomograms were highly consistent with the actual observed values while they had analysed expanding data set. In conclusion, the age and various stages (e.g., T-stage, N-stage and grade stages) are the independent risk factors for DM of osteosarcoma patients.

Similarly, Zhang et al. research teams proposed that nomograms could predict risk and prognostic factors for skeletal metastasis of pancreatic cancers while patient’s data diagnosed between 2010-2016 were analysed in the area under the curve (AUC), c-index, calibration curve, decision curve analysis, and Kaplan-Meier survival analysis to determine the predictive accuracy and clinical effectiveness of the nomograms. Thus, such nomogram models can be used to precisely predict the metastasis of cancer patients which can effectively assist clinicians for personalized treatment for cancer metastasis.

Similar to the above, Zhao et al. research group demonstrated that the plasma hyaluronic acid (HA) correlates with bone metastasis of lung cancer patients based on univariate and multivariate COX regression analysis where lung cancer patients were enrolled between 2017- 2020. This study also stated that after –two cycle chemotherapy (etoposide combined with cisplatin or carboplatin), HA could be an independent risk factor for overall survival and progression free survival. Moreover, dynamic alteration of HA may act as monitoring marker for chemotherapy for lung cancer patients.

Together these studies begin to identify new approaches for improved personalized medicine approaches in cancer patients. Better predictions of outcomes may help identify patients at higher risk for metastatic disease and that may respond more favorably to specific therapies. Below we will highlight the papers in this issue that are investigating novel therapeutic approaches.

## Therapy for the treatment of bone metastasis

While therapy has improved morbidity associated with tumor-induced bone destruction, therapies have been less successful for reducing or preventing tumor growth in bone. Thus, identifying new therapies remains a major focus. This collection of papers focused on identifying new approaches.

In Canuas-Landero et al. the University of Sheffield group lead by Dr. Ottewell investigated why adjuvant zoledronic acid (Zol) treatment has differential outcomes in patients with metastatic breast cancer. The AZURE clinical trial investigating adjuvant Zol treatment showed that only the post-menopausal women showed a survival benefit. This paper investigates the molecular mechanisms that may contribute to this differential response. They concluded that the inhibition of osteoclast mediated bone destruction was not altered by oestrogen concentration. However, the anti-tumor effect of Zol was highly variable between mouse strains and was more effective in mice with lower levels of oestrogen supplementation. While more work is needed to confirm, these results suggest that differential immune responses in pre- and post-menopausal women may contribute to the differential response of adjuvant Zol. Overall, this exciting study indicates that adjuvant Zol treatment may effectively reduce breast cancer metastasis in select patient populations. More studies to better define the groups that adjuvant Zol may most impact metastatic tumor burden and survival are needed, but the preliminary findings are encouraging.

The Straign et al., paper, “Targeting the BMP Pathway in Prostate Cancer Induced Bone Disease” investigated a small clinical dataset of bone metastatic prostate tumors by immunohistochemistry and demonstrated that BMP signaling was increased in prostate tumors associated with bone. Thus, they investigated a BMP antagonist (DMH1) in mouse models with and without tumor. Importantly, they showed that DMH1 does not impair normal bone nor is it associated toxicity. While early DMH1 treatment prevented tumor metastasis to bone, it did not inhibit tumor burden when treatment was delayed to 6 days post tumor inoculation. Similar to previous studies using TGF-β inhibitors, the timing of BMP inhibition is critical and limited to early stages of the metastatic process. As described in Canuas-Landero et al., better understanding of the effects of BMP inhibition is needed in order to carefully identifying the patients that may benefit from BMP inhibition. Additional studies to determine if BMP inhibition may show higher efficacy when paired with other therapeutic approaches may help identify novel approaches for reducing tumor burden in bone in metastatic prostate cancer patients.

Osteosarcomas remain a clinical challenge where molecularly targeted approaches have had limited success. Surgery and chemotherapy remain the primary therapeutic approach. Despite the rapid increase in immunotherapy use in many tumor types, these therapies have not been successful in bone associated tumors, including osteosarcoma. This case report, investigate the potential of combining chemotherapy and immunotherapy in osteosarcoma patients (Zheng et al., 2021). This study demonstrated high tolerance in the patient for chemotherapy and immunotherapy in combination with surgery as needed. This approach allowed for 2 years of well-controlled disease in this patient. As a case study, it is difficult to know how this approach may perform in a large clinical trial, but it provides another option in a tumor type with limited therapeutic options. Interestingly, this study measured changes in mutational burden over time in this patient. This gives interesting impact into how a tumor changes during the course of therapy and highlighted the high mutational burden in osteosarcoma, which lead to the failure of most targeted approaches. Thus, understanding these changes may lead to important discoveries of potential targets with low mutational frequency that may allow for implementation of molecularly targeted approaches in osteosarcoma.

These studies highlight the focus in cancer metastasis to bone on identifying new approaches to reduce or prevent tumor establishment in bone. Each of these studies focuses on different pathways and different tumors, but these studies can largely inform multiple tumor types. These studies each focused on refining “standard” approaches for the treatment of tumor-induced bone diseases, but add important details and potential options for therapeutic combinations to reduce tumor burden in bone while preventing bone loss or other toxicity.

## Influence of macrophages in bone metastasis

The final manuscript in this collection from Batoon and McCauley reviewed “Cross Talk Between Macrophages and Cancer Cells in the Bone Metastatic Environment”. This fantastic review detailed the role of different macrophage sub-types in bone metastasis from early metastasis from the primary site to colonization in bone, dormancy, and outgrowth. Altering macrophage responses is an active area of interest across the metastasis field and many other disease types (including hypertension, diabetes, bone disease, etc.). This paper describes potential novel therapeutic approaches that may reduce tumor associated macrophage number, alter macrophage polarization, or alter macrophage activity. This is a fast-moving field where combination therapies are likely to be key. Zoledronic acid and other therapies used commonly and being developed are known to interact with macrophages. Better understanding the role of the macrophage in disease may help make better therapeutic predictions and is likely to yield exciting new therapies.

## Calcium in cancer malignancy

In case of bone metastasis, hypercalcemia is also a common consequence since too much calcium is released to blood after bone decay. This elevated Ca2+ may further modulate the growth of cancer at primary and metastatic sites. While the use of bisphosphonates has largely reduced hypercalcemia, many groups have focused on understanding how calcium signaling, a major regulator of bone homeostasis, may impact tumor outcomes. In this context, Beasley et al. research team suggested that chronic exposure of triple negative breast cancer cells to high extracellular Ca2+ may decrease sensitivity of CaSR to Ca2+, but also stimulates cancer cell growth and migration with concomitant increase of a set of genes associated with malignant tumors including MAGEC2. Since calcium is found at high concentrations in the bone, understanding how Ca2+ may alter gene expression may also bring insight into tumor outcomes and drug responses.

## Conclusion

As the editors of this Research Topic, we hope you enjoy reading this collection as much as we did editing this collection. There are many exciting advances being made in tumor-induced bone diseases that are likely to have a high impact on patient outcomes over the next decade. We look forward to seeing the work presented here and new work continue to develop towards the goal of curing bone metastatic disease.

## Author Contributions

CM: Write and reviewed manuscript. JR: Write and edited manuscript. All authors contributed to the article and approved the submitted version.

## Conflict of Interest

The authors declare that the research was conducted in the absence of any commercial or financial relationships that could be construed as a potential conflict of interest.

## Publisher’s Note

All claims expressed in this article are solely those of the authors and do not necessarily represent those of their affiliated organizations, or those of the publisher, the editors and the reviewers. Any product that may be evaluated in this article, or claim that may be made by its manufacturer, is not guaranteed or endorsed by the publisher.
